# Synergy between Non-Thermal Plasma with Radiation Therapy and Olaparib in a Panel of Breast Cancer Cell Lines

**DOI:** 10.3390/cancers12020348

**Published:** 2020-02-04

**Authors:** Julie Lafontaine, Jean-Sébastien Boisvert, Audrey Glory, Sylvain Coulombe, Philip Wong

**Affiliations:** 1 Institut du Cancer de Montréal, CRCHUM, 900 Rue St. Denis, Montreal, QC H2X 0A9, Canada; julie.lafontaine.chum@ssss.gouv.qc.ca (J.L.); gloryaud@gmail.com (A.G.); 2 Plasma Processing Laboratory, Department of Chemical Engineering, McGill University, 3610 University Street, Montreal, QC H3A 0C5, Canada; 3 Département de Radio-oncologie, CHUM, 1051 rue Sanguinet, Montreal, QC H2X 3E4, Canada

**Keywords:** radiation therapy, non-thermal plasma, radio-frequency discharge, breast cancer, PARP-inhibitor, olaparib, DNA-damage

## Abstract

Cancer therapy has evolved to a more targeted approach and often involves drug combinations to achieve better response rates. Non-thermal plasma (NTP), a technology rapidly expanding its application in the medical field, is a near room temperature ionized gas capable of producing reactive species, and can induce cancer cell death both in vitro and *in vivo*. Here, we used proliferation assay to characterize the plasma sensitivity of fourteen breast cancer cell lines. These assays showed that all tested cell lines were sensitive to NTP. In addition, a good correlation was found comparing cell sensitivity to NTP and radiation therapy (RT), where cells that were sensitive to RT were also sensitive to plasma. Moreover, in some breast cancer cell lines, NTP and RT have a synergistic effect. Adding a dose of PARP-inhibitor olaparib to NTP treatment always increases the efficacy of the treatment. Olaparib also exhibits a synergistic effect with NTP, especially in triple negative breast cancer cells. Results presented here help elucidate the position of plasma use as a potential breast cancer treatment.

## 1. Introduction

Each year, more than two million women are diagnosed with breast cancer, the most frequent cancer in women [1]. Depending on the cancer subtype, patients’ prognosis differs along with the therapeutic approaches. For example, patients with a triple-negative breast cancer (TNBC), a subtype that does not express estrogen receptor (ER) and progesterone receptor (PR), and does not overexpress human epidermal growth factor receptor 2 (HER2), are associated with worsened prognosis [2,3]. Despite the identification of molecular subtypes (luminal, basal A, and basal B), and relative radioresistance of TNBC, adjuvant locoregional radiation therapy (RT) of the breast significantly reduces TNBC local recurrences [4,5], akin to all breast cancer subtypes.

A new perspective in cancer treatment has come with the use of non-thermal plasma (NTP). Briefly, NTP consists of a partially ionized gas which allows to convert electrical energy into chemical and thermal energy. Usually obtained by applying an electric field to a flowing gas [6], NTP is partially composed of electrons, ions and various reactive species [7]. In contact with air, plasma provides a remarkable tool to produce reactive oxygen and nitrogen species (RONS) that can interfere with cancer cells’ functioning and survival [8]. The anticancer capacity of NTP has been demonstrated in different cancer types (including breast cancer) in vitro [9,10,11,12,13,14], and in vivo [15,16,17,18,19]. In the clinic, one of the potential modalities of plasma treatment is its use in combination with surgery. For instance, NTP could be applied intraoperatively within the surgical cavity to potentially replace larger breast tissue resections, a method of treatment that does not necessarily require selectivity towards cancer cells [20]. As surgery is usually combined with other treatment modalities such as RT, it is therefore essential to investigate combination of NTP with other modalities.

As most studies on plasma oncology examined only a few cell lines to demonstrate the anticancer capacity of NTP, here we use a relatively high-throughput approach to characterize the sensitivity of fourteen human breast cancer cell lines to NTP. Hence, the aim of this work is twofold. First, to determine if the sensitivity of breast cancer cells to NTP is dependent on the molecular subtype. Second, to compare and evaluate the potential combination of NTP with RT and PARP (Poly (ADP-ribose) polymerase) inhibitor olaparib, two other therapies used in breast cancer. By comparing the plasma response of these cell lines to RT sensitivity, we observe a direct correlation between the efficacies of these two DNA damaging agents. Moreover, combining RT and plasma can result in a synergistic effect in a subset of cell lines. In addition, pretreatment with olaparib increased the efficacy of NTP in all tested cell lines. A synergistic effect was also measured for this combination, independent of the *BRCA1/2* status of the cell lines.

Precision oncology involves multidisciplinary approaches to define patient subgroups and adapt the use of various treatment modalities, either alone or in combinations, according to the disease’s sensitivities and patient’s needs. NTP can become a new member of the arsenal to treat patients affected by breast and other cancers. As NTP yield minor side effects [21,22], local application of NTP can reduce the local tumour burden and replace several fractions of RT to reduce RT-related side effects in a clinical setting.

## 2. Results

### 2.1. NTP Device and Experimental Setup

**Figure 1:**
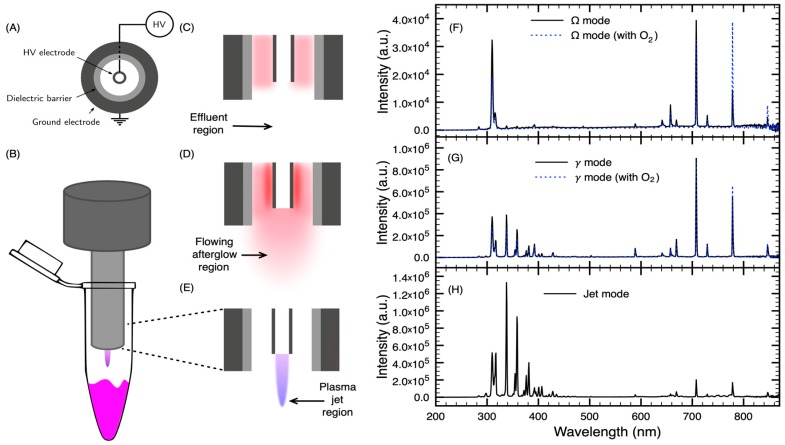
Experimental configuration and optical emission spectra of the different discharge modes with helium as the plasma-forming gas. (**A**) Simplified electrical circuit of the convertible plasma device. (**B**) Graphic representation of the treatment of cell suspensions in the jet mode. (**C**) Sketch of the convertible plasma device in the Ω mode. (**D**) Sketch of the convertible plasma device in the γ mode. (**E**) Sketch of the convertible plasma device in the jet mode. (**F**) Optical emission spectrum (OES) of the Ω mode without or with 2 mL min^−1^ of O_2_. (**G**) OES of the γ mode without or with 2 mL min^−1^ of O_2_. (**H**) OES of the jet mode.

Various plasma devices are used for research purposes in plasma oncology. Due to their versatility for applications both in vitro and *in vivo*, plasma jets are most often used [23]. Figure 1 presents a sketch of the convertible plasma device and the experimental setup used in our experiments. This convertible plasma device allowed us to perform treatments in three different discharge modes [24]. The electrical configuration is illustrated in Figure 1A. The electric field is supplied by a high-voltage capillary electrode mounted coaxially inside a cylindrical ground electrode. A dielectric barrier lies between the annular gap and the ground electrode. Using an excitation frequency of 13.56 MHz and a flow of helium, the Ω, *γ* and jet modes (Figure 1C–E, respectively) can be generated. In the Ω mode, the plasma is sustained within the device and only plasma effluents can reach the treatment zone (Figure 1C). In the *γ* mode, a higher power density is injected into the plasma and a flowing afterglow is produced at the tip of the nozzle (Figure 1D). In the jet mode, no plasma is formed within the annular gap between the dielectric barrier and the high-voltage electrode, but it is formed at the tip of the nozzle (Figure 1E).

As the high-voltage electrode is hollow, a secondary gas can be injected in the effluent zone of the Ω mode or the flowing afterglow in *γ* mode. Addition of O_2_ in rare gas NTPs is a reliable way to increase the production of RONS that can influence the anticancer capacity of the treatment [25,26]. As shown in Figure 1F,G, injection of O_2_ in the high-voltage electrode allows to selectively enhance the atomic oxygen line O (3^5^P→3^5^S) (center wavelength at 777.5 nm). As optical emission spectroscopy (OES) does not allow to probe non-fluorescent atoms and molecules, the observation of this oxygen line can act as an indicator of the production of RONS within the plasma effluent or afterglow region.

### 2.2. Influence of the Discharge Mode on the Cytotoxicity of the Treatment

One aim of the present work is to determine if a subgroup of breast cancers could be more susceptible to plasma treatment. In order to address this, a panel of fourteen cell lines that contained representatives of each breast cancer subtype was used. Characteristics of theses cell lines are presented in Table 1.

**Figure 2:**
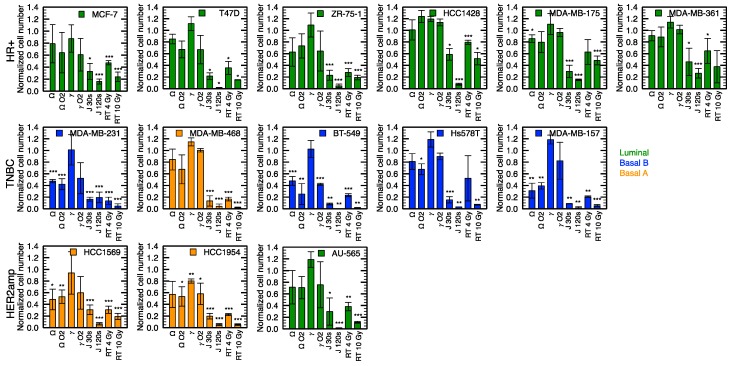
Comparison of the efficiency of different treatments (see Table 2 for experimental conditions) on a panel of breast cancer cell lines using proliferation assays. Hormone receptor positive (HR+), Triple negative breast cancer (TNBC) and HER2 amplified (HER2amp) define the receptor status of cell lines and the color code refers to the molecular subtype. The Ω and *γ* modes (4 and 2 min) were compared with and without the injection of 2 mL min^-1^ of O_2_ in the high-voltage electrode. Two doses were compared for the jet mode (30 and 120 s) and for radiation therapy (4 and 10 Gy). Error bars represent the standard deviation over three independent experiments. * *p* < 0.05, ** *p* < 0.01, *** *p* < 0.001 with respect to the control.

The convertible plasma device used in this study allows to treat cells using three different discharge modes. As we previously reported, helium gas flow alone (without applied power) does not produce a cytotoxic effect in any of the conditions selected for this work (Table 2) [24]. However, all discharge modes show a cytotoxic effect. In fact, depending on the selected discharge mode, various treatment times are required to achieve the same antiproliferation capacity [24]. This efficiency is confirmed here with a larger number of cell lines (Figure 2). In comparison with treatment of 4 and 2 min of the Ω and *γ* modes respectively, the jet mode requires less time to treat cells, with a more intense effect reached with only 30 s of treatment for all cell lines. Proliferation assays revealed plasma sensitivity across all cell lines with normalized cell number reduction ranging from 0 to 70% for Ω mode and 40% to 90% for jet mode. Only the HCC1954 cell line responded to the *γ* mode, with 20% of normalized cell number reduction after treatment. Importantly, the efficacy of all NTP modes increases with treatment time, akin to drug or RT dose response curve. Time response curves for the jet mode are shown in the next section.

For comparison with plasma treatments, RT was used as a standardized reference for cytotoxic sensitivity of the different cell lines. A 4 Gy reference dose of RT tends to be more cytotoxic than both the Ω and *γ* modes in all cell lines. Cytotoxicity of the jet mode is similar to the cytotoxic effects of RT and in some cases greater than the response to 10 Gy (e.g., *p* < 0.01, comparing 120 s of jet mode with 10 Gy of RT in HCC1428). Additionally, the most radioresistant cell lines, HCC1428 and MDA-MB-175-VII, were sensitive to the jet mode using a sufficient dose of 120 s (*p* < 0.001).

**Figure 3:**
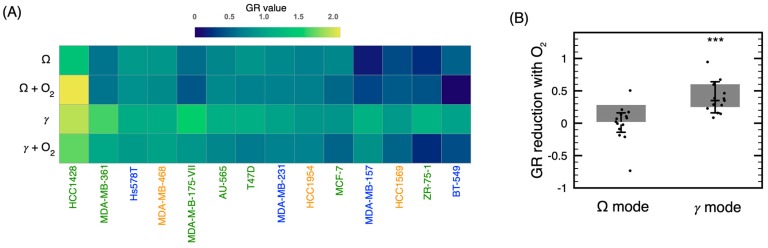
Influence of O_2_ on the antiproliferative capacity of NTP. (**A**) Heat map of GR values (low GR values indicate high treatment efficacy) of treatments in the Ω and *γ* modes with and without the addition of O_2_ in the high-voltage electrode. Classification of the cell lines was selected using the values of GR averaged over all discharge modes. Cell line color classification: Luminal (Green), Basal A (Orange) and Basal B (Blue). (**B**) Box-and-whisker plot of the variation of GR value (GR value without O_2_ minus GR value with O_2_) in the Ω and *γ* modes. *** *p* < 0.001 with respect to the GR without O_2_.

Another feature of the device is the possibility to inject a secondary gas directly into the plasma effluent or afterglow (via the hollow high-voltage electrode). Figure 2 indicates that, in the Ω mode, for some cell lines, O_2_ slightly increased the antiproliferative capacity of the treatment, while for other cell lines, the antiproliferative capacity remained unchanged. On the other hand, with the *γ* mode, addition of O_2_ to the treatment tends to improve the antiproliferative capacity of plasma for all cell lines. However, the sensitivity to the addition of O_2_ varies significantly between different cell lines. Comparison of the antiproliferative effect of O_2_ is illustrated in Figure 3, where GR values (see Section 2.3 and Section 4.4 for details on GR values) are displayed in a heat map and GR variation in a box-and-whisker plot.

Results shown in Figure 3A are in good agreement with those of Figure 2. Indeed, the antiproliferative effect of Ω and *γ* modes is enhanced by the injection of O_2_ in the high-voltage electrode. However, from the use of GR values, it is possible to quantify the overall sensitivity of the different cell lines. As shown by Figure 3B, the variation of GR values by injecting O_2_ in the high-voltage electrode is almost inexistent in the Ω mode (*p* > 0.5 with *t*-test) but is significant in the *γ* mode (*p* < 0.001 with *t*-test). Even if the injection of O_2_ in our NTP device is performed downstream (in the effluent or the flowing afterglow region) rather than in the plasma itself, the antiproliferative capacity can benefit from the injection of O_2_. This feature is in agreement with other studies reporting on the anti-cancer effect of O_2_ addition to plasma [28,29] but highlights the potential advantage of downstream injection for NTP optimization.

### 2.3. Sensitivity of Breast Cancer Cell Lines to NTP Correlates with RT

**Figure 4:**
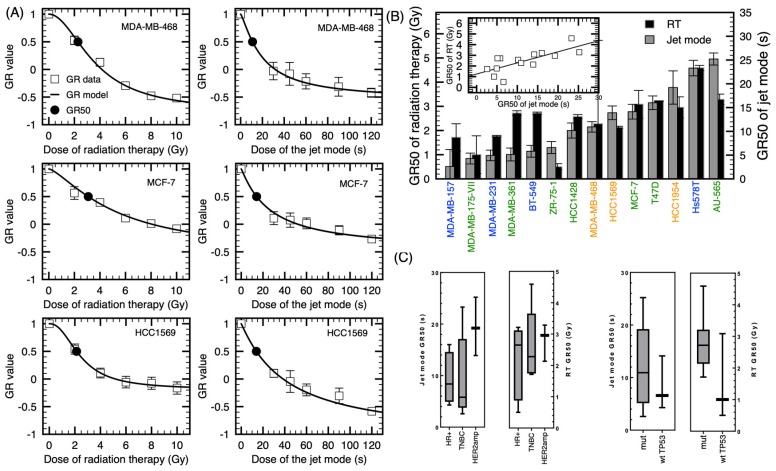
Dose response and sensitivity of different cell lines to the jet mode and radiation therapy. (**A**) Dose-response curves with the theoretical curve obtained from a log-logic fit, with radiation on the left and plasma treatment on the right. Error bars represent the standard deviation over three independent experiments. (**B**) Calculated GR_50_ of radiation therapy and jet mode. Pearson’s correlation plot of the GR_50_ of the jet mode and GR_50_ of radiation therapy (*p* < 0.005). (**C**) Box-and-whisker plots of GR_50_ classified according to the receptor status and TP53 mutation.

Classification of breast cancer cell lines according to their sensitivity to NTP (jet mode) and to RT is shown in Figure 4. In order to classify the cell lines in terms of their sensitivity, the growth rate (GR) metrics was utilized [30]. Based on a dose-induced GR inhibition, the GR metrics allows to generate dose-response curves that are not influenced by the division rate, and therefore the derived sensitivity is more representative of the genotype of each cell line. This is particularly relevant since our output was measured after six days of incubation and the reported range of doubling time for these cell lines varies from 1.3 to 4.6 days [31]. Given that we quantify cell numbers to determine NTP efficacy, we need to mitigate this confounding factor. As expected, applying the GR metrics to our data slightly changed the classification of the cell lines according to their sensitivity (see Appendix B). Three representative curves of the GR dose-response with the sigmoidal fit based on the number of cells at the beginning of the experiment using a fixed interval approach are shown in Figure 4A. For the more responsive cell lines, MDA-MB-157, MDA-MB-175VII and MDA-MB-231, an exposure time lower than 5 s was required to reach the GR_50_ (Figure 4B). For other cell lines, the exposure time never needed to exceed 30 s. Interestingly, there is a strong correlation between the GR_50_ of RT and the GR_50_ of the jet mode, with a Pearson’s coefficient of correlation of *p* < 0.005 (Figure 4B). This suggests that the reactivity of cells to plasma treatment could be extrapolated according to their radiosensitivity.

We then determined if sensitivity to plasma treatment can be correlated to a few different clinical aspects of breast cancer. We grouped the cell lines into their different receptor status subtypes (HR+, TBNC and HER2amp). In Figure 4C, although the mean GR value of HER2amp group seems higher, we did not find a statistical difference between the groups. Grouping the cell lines according to their p53 status did not exhibit a significant correlation either. Again, sensitivity of cell lines classified according to their receptor status subtypes and p53 status are similar for NTP and RT. These results showed that even if all the tested cell lines were sensitive to plasma treatment, we did not point out a particular subtype of breast cancer which could be more sensitive to NTP.

### 2.4. Radiosensitization of Breast Cancer Cell Lines with NTP

**Figure 5:**
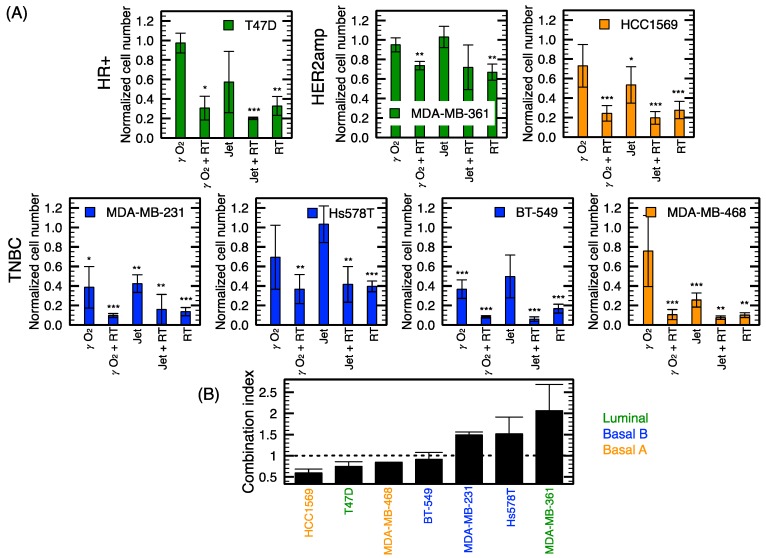
Combination of RT (4 Gy) with NTP (jet mode for 10 s). (**A**) Normalized cell numbers for different combination of treatments of a subpanel of cell lines. (**B**) Combination index (CI < 1: synergistic, CI = 1: additive, CI > 1: antagonist) of the jet mode and radiation therapy. Error bars represent the standard deviation over three independent experiments. Hormone receptor positive (HR+), Triple negative breast cancer (TNBC) and HER2 amplified (HER2amp) define the receptor status of cell lines and the color code refers to the molecular subtype. * *p* < 0.05, ** *p* < 0.01, *** *p* < 0.001 with respect to the control.

It is known that NTP can produce RONS [32], which can lead to either single- or double-strand DNA breaks (SSB or DSB) [33]. We previously confirmed the ability of plasma treatment to induce DNA damage [24]. We then hypothesized that the combination of NTP with another DNA damaging agent could increase the level of DNA damage. As half of all cancer patients will receive RT [34], it was chosen as a DNA damaging treatment to combine with NTP. The combination of NTP with RT is shown in Figure 5. A subgroup of cell lines was used to determine the impact of combining DNA-damaging agents, focusing on TNBC. As described in Section 4, NTP was immediately followed by RT.

Figure 5 shows that both NTP conditions tested tend to be combining efficiently with RT. We also demonstrated that a jet treatment as short as 10 s is sufficient to produce a cytotoxic effect in most cell lines. The cellular response to the jet mode alone for 10 s of exposure is similar to the *γ* mode with O_2_, but both displayed lower antiproliferative capacity than 4 Gy alone (Figure 5A). For a better appreciation of the efficacy of the combined treatment, the combination index (CI) was evaluated according to the Chou-Talalay method [35,36,37]. CIs were calculated for the combination of the jet mode with RT as dose-response curves were established for these treatment modalities (see Figure 4A).

Figure 5B shows the classification of cell lines according to the CIs and reveals a synergistic effect for four out of seven cell lines. Only two of the four TNBC cell lines (MDA-MB-468 and BT-549) showed a synergistic effect.

### 2.5. Olaparib Influence on NTP Growth Inhibition and DNA Damage Potential

Cancer therapy using radiation or other DNA-damaging agents is based on the susceptibility of cancer cells to genomic instability [38]. Therefore, targeting DNA repair components to sensitize cells to genotoxic stress is a promising avenue to improve plasma treatment. An interesting agent to combine with NTP for breast cancer treatment is the PARP-inhibitor olaparib, a clinically approved treatment. We purposely chose a low dose of olaparib (2 µM) in order to minimize the effect of the drug itself on cell growth. This dose was also commonly used in combination assays [39,40], and is lower than the IC_50_ according to the literature [41,42]. We also selected the time of exposition to the jet mode at 10 s.

**Figure 6:**
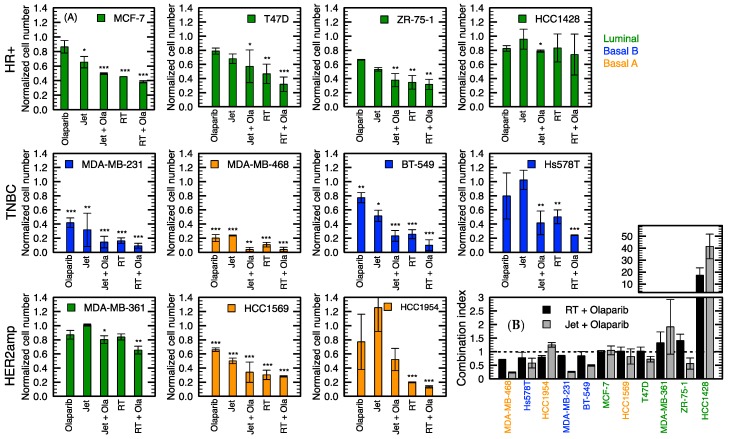
Combination of the jet mode (10 s) with olaparib (2 μM) for a subpanel of cell lines. (**A**) Normalized cell numbers for different combinations of treatments. (**B**) Combination index (CI < 1: synergistic, CI = 1: additive, CI > 1: antagonist) of the jet mode (10 s) and radiation therapy (4 Gy) with olaparib (2 µM). Error bars represent the standard deviation over three independent experiments. * *p* < 0.05, ** *p* < 0.01, *** *p* < 0.001 with respect to the control.

As shown in Figure 6, even 2 µM of olaparib alone had an effect on cell growth, especially for MDA-MB-468, which presented the highest sensitivity to the drug (more than 60% of inhibition, *p* < 0.001). Among the cell lines used, only HCC1569 and MDA-MB-361 contained a BRCA2 mutation. In those cell lines, only 40% (*p* < 0.001) and 20% (*p* > 0.05) of growth inhibition was observed, respectively, with olaparib alone. Indeed, the dose used was not enough to reach the synthetic lethality expected in BRCA mutants. Olaparib treatment alone resulted in growth inhibition ranging from 0% to 40% in other cell lines.

In all cell lines, combination with olaparib tends to improve the cytotoxic effect of plasma. Interestingly, CI < 1 for seven out of eleven cell lines, demonstrating a synergistic effect of olaparib and plasma (Figure 6B). This was also true for the combination of RT and olaparib. For the few cell lines with CI > 1, including the BRCA2-mutation-bearing MDA-MB-361, the low dose of jet mode might be responsible for this antagonist combination. This combination could possibly be improved with a higher dose of plasma. Moreover, with every TNBC cell lines (MDA-MB-231, MDA-MB-468, BT-549 and Hs578T), the cytotoxic effect of the dual therapy tends to be better than radiation alone. This implies that plasma treatment, which is considered as a soft treatment in terms of side effects [21], in combination with olaparib, can be used to give the same response as 4 Gy with potentially fewer side effects.

DNA damage can be visualized through the activation of DNA repair pathways. We used the detection of DNA-damage-associated foci phosphorylation of H2AX (γH2AX) to characterize the effect of our combination at the molecular level.

**Figure 7:**
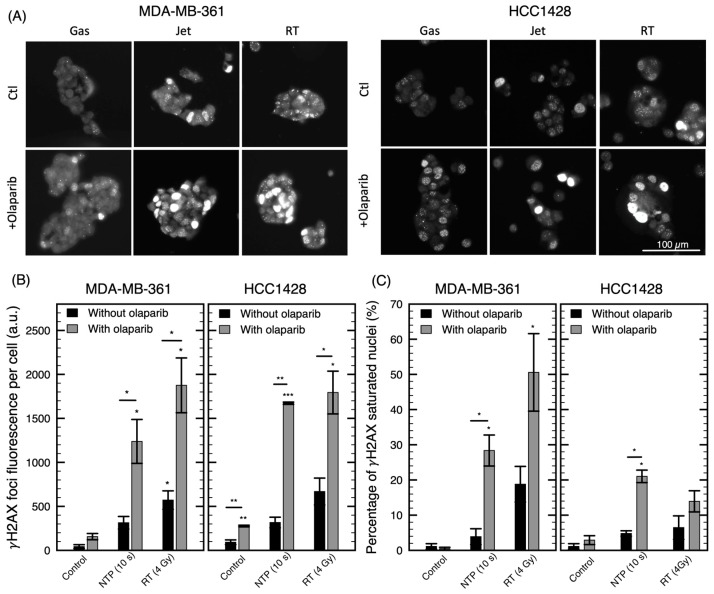
Visualization of DNA damage for a combination of olaparib with NTP or RT treatments. Immunofluorescence of γH2AX foci 24 h after treatment with gas (10 s), jet (10 s), RT (4 Gy) alone or in combination with olaparib (2 µM). (**A**) Example pictures of γH2AX for MDA-MB-361 and HCC1428 cell lines. (**B**) Total fluorescence of γH2AX foci per cell (MDA-MB-361 and HCC1428). (**C**) Percentage of nuclei with fluorescence of γH2AX foci covering the complete nucleus. Error bars represent the standard deviation over two or three pictures containing an average of 30 nuclei. * *p* < 0.05, ** *p* < 0.01, *** *p* < 0.001 with respect to the control or the equivalent condition without olaparib.

As expected, in Figure 7, we observed an increase in the number of foci with the jet or radiation alone compared to the control, confirming the induction of DNA damages by the oxidizing agents. Olaparib alone is also known to cause an increase in γH2AX foci in responding cancer cells [43]. We observed this increase in the two cell lines presented here (*p* < 0.01 for HCC1428). In addition, pretreatment with olaparib further increased the number and intensity of foci following a treatment by jet mode and RT (*p* < 0.05). Interestingly, in some conditions, a strong pan-nuclear staining can be observed in a portion of the cells. This pan-nuclear phosphorylation of H2AX signal may suggest cells potentially going through apoptosis following treatment [44,45]. According to Figure 7C, this is especially the case for combination treatments (up to 50% of the cells are exhibiting pan-nuclear staining).

These results indicate that the effect of plasma on cancer cells can be improved by a combination with a DNA repair inhibitor such as olaparib. This reinforces the notion that the ability of NTP to induce cytotoxic effects occurs through DNA damage (whether SSB or DSB), similar to radiation.

## 3. Discussion

In this study, we compared the sensitivity to NTP with RT, a conventionally used modality in breast cancer treatment, on a large set of common breast cancer cell lines. A constraint to large-scale comparison of NTP sensitivity across cell lines is the heterogenous proliferation rates of different cell lines. Our method to quantify cell response to NTP was based on the normalized cell number 6 days post-treatment. Therefore, systematic variations in cell division time will affect relative response metrics such as IC_50_ (treatment condition resulting in 50% relative viability) [30]. To overcome this confounder, the Growth Rate (GR) metrics was used to quantify cellular response to treatments. For instance, MDA-MB-175VII, that was not particularly sensitive to either NTP or RT in terms of normalized cell number (Figure 2), was found to be one of the most sensitive cell lines when GR values were used (Figure 4). As the doubling time of MDA-MB-175VII is 5.5 d (see Table A1 in Appendix B), this cell line provides the typical situation where IC_50_ is confounded by its slow cell division rate. The GR method is commonly used in a variety of open access databases compiling the drug sensitivity of different cell lines. The Library of Integrated Network-based Cellular Signatures (LINCS) [46] and PharmacoDB [47] are two of them.

Beside the use of fourteen cell lines, three NTPs, described previously [24], have been used in the present study. The longer treatment time required by the Ω and *γ* modes (in comparison to the jet mode) to reach a similar antiproliferative capacity turned out to be respected for all cell lines. As previously reported [28,29], addition of O_2_ to rare gas NTP increases the cytotoxicity of plasma. However, in the present case, O_2_ is not injected in the plasma per se, it is rather injected in the effluent region (in the Ω mode) or in the flowing afterglow (in the *γ* mode). As shown in Figure 2 and Figure 3, over all cell lines, the influence of O_2_ is significant in the *γ* mode. This suggests that injection of O_2_ in the flowing afterglow is sufficient to enhance the production of RONS, a fact in agreement with the enhancement of the O (3^5^P→3^5^S, 777.5 nm) atomic emission line in Figure 1 (also reported in similar conditions [48]). The lack of effect of O_2_ in the Ω mode could be attributed to the low energy present in the plasma effluent. However, as Figure 1F clearly shows an enhancement of the 777.5 nm atomic emission line with injection of O_2_, the mitigate effect of O_2_ could indicate that the 777.5 nm atomic line is not a good indicator of plasma antiproliferation capacity (at least with downstream O_2_ injection) or it could be simply due to the NTP dose that is too low to significantly impact cell proliferation. This will be the subject of further investigations.

We previously reported that plasma can induce DNA damage in MDA-MB-231 cell line and drive the cells to undergo mitotic catastrophe [24]. Here, in Figure 7, we showed that DNA damage is also induced in the two additionally investigated cell lines. These findings suggest that DNA repair inhibitors may increase the efficacy of NTP as observed with other DNA damaging treatments [49,50]. Similarly, increasing the number of insults by combining two DNA damaging agents is known to improve cytotoxicity [51,52]. In this work, a combination of NTP with olaparib or RT was investigated to address these two options. In both cases, most cell lines were found to benefit from the combination. In fact, in some conditions, NTP yields a synergistic combination with RT or olaparib.

Using the same experimental method to investigate the sensitivity of cell lines to NTP and RT allowed to establish a strong correlation between both modalities’ antiproliferative capacity. This correlation highlights the similarity in the mechanisms of action of these modalities. In addition, using the same procedure, the combination of RT and NTP have also been tested. In agreement with previous work by Lin et al. [53] in other types of cancer cell lines, greater growth inhibition was found with the combination compared to NTP or RT alone. An observation in support of the increased in DNA damage previously reported with the combination [53]. Here, RT and NTP’s combination was addressed in a subset of cell lines, focusing on TNBC since they represent the subtype clinically more complex to treat and present higher local recurrence rates. In half of the TNBC, the combination synergistically increased the efficacy of the treatment. It was interesting to note that for the Basal A molecular class, the combination clearly improved NTP treatment. In Figure 4, Basal A subtype classified among the less responsive group. Nevertheless, adding RT synergistically improved their response. For other cell lines, we propose that the dose of 4 Gy alone was found to induce important cytotoxic effect, which could have hindered the potential benefit of its combination with NTP. The exposition time for the jet mode was also a fixed dose for every cell line. Since we determined the GR_50_ for the jet mode and RT, the doses required for combination could be better defined for each cell line in future experiments. Results from the current study supports the fact that the application of NTP in combination with RT may be complementary and a viable clinical strategy for further exploration.

Olaparib is approved by the US Food and Drug Administration (FDA) as a therapy for select breast, ovarian and prostate cancers, for which plasma treatment is also being evaluated in vitro [19,33,54,55]. In breast cancer, olaparib is approved for the treatment of patients with germline deleterious mutations in BRCA, HER2-negative metastatic breast cancer who had previously received chemotherapy. Our results present further evidence that olaparib can be beneficial to more patients, independently of the BRCA status, when used in combination with other DNA damaging agents. Within the context of NTP, concurrent olaparib could improve the local control of the disease.

In our experiment, we chose to target PARP, which is mainly involved in SSB repair. Unresolved SSB will generally evolve in DSB, and direct induction of both types of DNA damage by RONS is probably the main mechanism of genotoxic stress by plasma treatment [33]. In the same line of thought, Masur et al. [56] used gemcitabine, a nucleoside analogue, that affects DNA synthesis and repair. They reported that combination with plasma allows to decrease the dose of Gemcitabine required to observe a cytotoxic effect. Gemcitabine is also a drug indicated in advanced stages of breast, pancreatic, ovarian, and non-small cell lung cancer [57]. Molecular targeted agents such as cetuximab, an antibody that targets epidermal growth factor receptor (EGFR), have also been shown to increase plasma efficacy when used in combination against cancer cells [58]. Therefore, it is expected that targeting other DNA-repair or proliferation pathway components in combination with plasma therapy will achieve better response rates than plasma alone. Thereby, all combinations displayed here and previously reported suggest a promising advance for the treatment of breast cancer. Indeed, gemcitabine [59] and radiation [53], have already been tested in vivo in combination with NTP, and demonstrated increased response over a single treatment. A better understanding of the downstream molecular impact of plasma treatment will open novel routes to identifying compounds that can improve NTP efficacy or vice versa.

In characterizing the relative sensitivity of a large set of publicly available breast cancer cell lines, this study built a platform to further explore the cellular mechanism of action of plasma. As these cell lines’ genomes have been sequenced [60,61,62] and rendered publicly available, the next step will be to investigate the molecular signature from sensitive and resistant cell lines to non-thermal plasma treatment. We hope such an analysis will help reveal candidate pathways involved in plasma’s mechanism of action, a current challenge in the field of plasma medicine.

## 4. Materials and Methods

### 4.1. Cell Culture

Breast cancer cell lines Panel 1 (AU-565, BT-549, HCC1428, HCC1569, HCC1954, Hs578T, MCF-7, MDA-MB-157, MDA-MB-175-VII, MDA-MB-231, MDA-MB-361, MDA-MB-468, T47D, ZR-75-1) was purchased from the American Type Culture Collection (ATCC, Manassas, VA, USA). Cells were grown following ATCC recommendations (DMEM, RPMI1640 or L15 supplemented with 10% or 20% foetal bovine serum (FBS), with or without insulin, with 1% penicillin-streptomycin (Pen-strep). Cells were incubated at 37 °C with or without 5% CO_2_ according to ATCC recommendations. Cells were used at a low passage number (lower than 25) upon reception from ATCC in order to maintain their parental phenotype and genotype. Genomic characterization of all investigated cell lines is publicly available [62]. This includes data regarding all the cell lines’ genetic, RNA splicing, DNA methylation, histone H3 modification, microRNA expression and reverse-phase protein array data.

### 4.2. Plasma Device

The convertible plasma device used for the treatment of cells in suspension was described elsewhere [24]. In brief, the device can be categorized as a plasma jet using a coaxial electrode configuration (Figure 1A). A power system (model 1312 Cesar, Dressler, Stolberg-Vicht, Germany equipped with a 57020137-00D Navio matching network, Advanced Energy, Fort Collins, CO, USA) delivers a 13.56 MHz excitation waveform to a hollow high-voltage electrode located on the axis of the device. A fused silica tube is located between the 1 mm annular gas gap and the outer shell ground electrode. Injecting helium through the annular gas gap or within the high-voltage electrode allows the maintenance of an electrical discharge in various modes (Figure 1C–E). Since theses discharge modes have different properties (volume, electron energy, electron temperature, etc.), it is important to validate if they produce similar response on different cancer cells. To reduce the number of experimental parameters investigated, a few parameters were fixed for this work. This is the case of the injected power and flow rate of the plasma-forming gas. The experimental conditions used for the production of NTP are summarized in Table 2. These conditions were chosen to yield a similar cytotoxicity in breast cancer cell lines between the different modes. This choice was based on a previous work that shown that the same antiproliferation effect is observed when cells are exposed to about 25 s of jet mode, 2 min of *γ* mode or 4 min of Ω mode [24].

The optical emission of the discharge was collected by an optical fibre for emission spectroscopy (Figure 1). Optical emission spectra are sampled downstream of the nozzle collecting light in the axis perpendicular to the gas flow to avoid collecting light from inside the device. The tip of an optical fibre (300–1100 nm with a 600 µm core diameter and a length of 1 m, Ocean Optics, Dunedin, FL, USA) is positioned 3 mm away from the exit nozzle. The optical fibre is connected to a spectrometer system (from 200 to 850 nm) through a 100 µm slit (Flame-S equipped with an ILX-511B Sony detector, and a 600 line mm^−1^ grating blazed at 300 nm with a resolution about 1.5 nm, Ocean Optics, Dunedin, FL, USA). The optical emission spectroscopy system was corrected by its complete response curve using an Intellical lamp (Princeton Instruments, Trenton, NJ, USA) above 400 nm and a 900 W tungsten lamp (Oriel, Irvin, CA, USA) below.

### 4.3. Radiation Therapy and Plasma Treatment

Cell suspensions were prepared in DMEM containing pyruvate supplemented with 10% FBS and 1% Pen-strep. A fixed volume of 400 µL was dispensed in 1.5 mL microtubes for plasma or radiation treatment. Cell concentration was adjusted according to the different cell lines (ranging from 50 000 to 200 000 cells/mL). Plasma treatment was performed by positioning the nozzle of the device inside the tube at a constant distance of 5 mm from the surface of the media. Figure 1B shows a sketch of the convertible plasma device during a treatment of cells in suspension. RT was performed using a caesium-137 source (Gammacell 3000 Elan, Best Theratronics, Ottawa, ON, Canada) for indicated doses (from 2 to 10 Gy). After treatment, 50 µL of the cell suspension from the microtube was transferred to a 48-well plate containing 200 µL of fresh media (specific for each cell line, according to ATCC). A condition with gas flow alone (without electric field) was included as a control to ensure that gas flow by itself did not have an effect on cell growth. Each experiment was performed three times. For the experiment using pyruvate, a final concentration of 1 mM was added to the RPMI or DMEM (without pyruvate) media.

### 4.4. Proliferation Assay

Cells seeded in a 48-well plate were fixed at day 6 after treatment with a crystal violet solution (20% methanol, 0.5% crystal violet). Fixed cells were then stained with DRAQ5 in PBS, washed and scanned with the Odyssey imaging system (LI-COR Biotechnology, Lincoln, NE, USA). A dilution curve of various cell numbers stained by DRAQ5 was prepared for each cell line in each experiment. DRAQ5 signal in each well was compared to the dilution curves to quantify the cell numbers in each well (normalized cell numbers).

Dose-response curves were calculated using the GR inhibition metrics [30]. This metrics can be calculated using *x*(*c*) the cell number of the treated sample at concentration *c*, *x*_0_ the cell number at the time *t* = 0 s (i.e., when the treatment is performed), *x_ctl_* the cell number of the control sample at the same time as *x*(*c*), according to the following equation

(1) $$GR\left(c\right)=2^{\frac{\log_{2}\left(x\left(c\right)/x_{0}\right)}{\log_{2}\left(x_{ctl}/x_{0}\right)}}-1$$

To use the GR metrics with our proliferation assay, *x*_0_ value were inferred from the number of cells plated, fixed at 18 h and stained with DRAQ5. GR_50_ for jet and RT were obtained for different doses in order to obtain dose-response curves.

### 4.5. Peroxide Detection

For hydrogen peroxide detection, cell suspensions were centrifuged after plasma treatment, and the supernatant was collected and processed immediately. Pierce^TM^ Quantitative Peroxide Assay Kit (Cat. no. 23280, Thermo Scientific, Saint-Laurent, QC, Canada) was used according to the manufacturer’s instructions. The optical density at 595 nm was measured with the Spark multimode microplate reader (TECAN, Männedorf, Switzerland).

### 4.6. Live Cell Imaging System

Live cells were followed using IncuCyte S3 Live-cell Imaging System (Sartorius, Göttingen, Germany), using a 10× objective. During the incubation period, cells were maintained in 48-well plates with 250 μL of medium per well. Only 20% of this medium was carried from the treated microtube as described in Section 4.3. Propidium iodide (PI) staining was used for cell death visualization and was added directly to the media (final concentration 1 µg mL^−1^) just before imaging.

### 4.7. Combination Treatment

For the combination with PARP-inhibitor olaparib (AZD2281, Selleckchem, Houston, TX, USA), cells were trypsinized, diluted to the appropriate concentration and pretreated with 2 μM of olaparib for 2 h before plasma treatment (or radiation). Following plasma treatment, 50 μL of the treated cells were transferred to a 48-well plate containing 200 μL of fresh media. Cells pretreated with olaparib were also maintained in media containing 2 μM of olaparib during the six days of incubation. For the combination with radiation, the plasma treatment was performed first and then cells were exposed to 4 Gy of radiation, within 30 min. The Chou-Talalay method [36] for drug combinations was used to calculate the CI for the combinations of plasma and radiation or plasma and olaparib. Dose-response curves were plotted for RT and olaparib treatment.

### 4.8. Immunofluorescence

After treatment, cells were plated in 8-well chamber slides and fixed 24 h later with 10% formalin for 5 min. Cells were permeabilized with 0.25% Triton for 10 min, incubated in blocking solution (4% Donkey serum, 1% BSA, PBS) for 1 h and with the primary antibody overnight at 4 °C. Cells were washed and incubated with the secondary antibody for 1 h at room temperature and then washed again. Prolong containing DAPI was used for slide mounting and images were obtained using a Zeiss Observer Z1 microscope (400×, Carl Zeiss, Oberkochen, Germany). Primary antibody used was phospho-H2AX (Ser139) (1:2 000 dilution; cat. No. clone JBW301) and secondary antibody was Alexa fluor-488 (1:750).

### 4.9. Statistical Analysis

Student’s *t*-test were used to compare different treatments with the control, to compare the effects of NTP between different breast cancer subtypes and to compare the influence of O_2_ on the antiproliferative effect of NTP in the *γ* mode. Pearson’s correlation test was performed to determine the correlation of the antiproliferative effect of NTP and RT. Data analysis, microscopy image analysis and statistical analysis were performed using homemade codes with Mathematica 10 (Wolfram, Champaign, IL, USA).

## 5. Conclusions

In this study, we were able to classify a large panel of breast cancer cell lines according to their sensitivity to NTP in three different discharge modes. Cell lines that are more sensitive to a discharge mode are also found to be more sensitive to other discharge modes. In addition, the Ω and *γ* modes were compared when a small concentration of O_2_ is injected via the high-voltage electrode. Unlike previous works were O_2_ is injected within the plasma itself, our plasma device allows the injection of O_2_ downstream of the plasma. In the *γ* mode, O_2_ is in contact with the flowing afterglow and a significant increase of the antiproliferative capacity of the plasma occurs. This effect was observed on all cell lines, and highlights the potential benefit on cancer treatment of the precise control of gas composition via downstream injection.

One of the aims of this work was to determine if a particular subtype of breast cancer could be particularly sensitive to NTP. Classification was selected with respect to their molecular subtype (i.e., luminal, basal A and basal B) and to their receptor status (i.e., HER2amp, TNBC, HR+). Using the GR_50_ to compare cell lines classified in the aforementioned subgroups, no subgroup of cell lines could be identified as strongly sensitive or resistant to NTP. Hence this highlights the potential clinical benefit to a majority of patient, should NTP be used intraoperatively to treat the tumour bed after tumour resection.

Comparing the sensitivity of cancer cell lines to NTP with their sensitivity to RT, a good correlation was found: cell lines more sensitive to RT are also more sensitive to plasma. Combination of NTP and RT was also found synergistic on a subpopulation of cell lines. These results suggest that adding a relatively low dose of NTP to a patient’s therapeutic plan could allow to reduce the dose of RT required, therefore minimizing side effects without compromising efficacy. In addition, we demonstrated for the first time that NTP can synergistically be combined with olaparib, a PARP inhibitor. Olaparib being more and more used in the clinic, its role as a NTP sensitizer is a very important feature towards the eventual position NTP could hold in the arsenal of breast cancer treatment.

## Figures and Tables

**Table 1:** Panel of breast cancer cell lines with molecular subtype, receptor status and list of mutations [27]. Molecular subtypes are classified as Luminal (green), Basal B (blue) and Basal A (orange).

Cell Line	Molecular Subtype	Receptor Status	Mutation Summary
AU-565	Luminal	HER2amp	TP53, MLL3
BT-549	Basal B	TNBC	TP53, PTEN
HCC1428	Luminal	HR+	TP53
HCC1569	Basal A	HER2amp	TP53, MLL3, BRCA2, PTEN
HCC1954	Basal A	HER2amp	TP53, PIK3CA
Hs578T	Basal B	TNBC	TP53
MCF-7	Luminal	HR+	PIK3CA, GATA3
MDA-MB-157	Basal B	TNBC	TP53, MAP3K1
MDA-MB-175-VII	Luminal	HR+	MLL3
MDA-MB-231	Basal B	TNBC	TP53
MDA-MB-361	Luminal	HR+	TP53, PIK3CA, BRCA2
MDA-MB-468	Basal A	TNBC	TP53, MLL3, PTEN
T47D	Luminal	HR+	TP53, PIK3CA, MLL3
ZR-75-1	Luminal	HR+	PTEN

**Table 2:** Summary of the NPT conditions used in this work. In the Ω and the γ modes, helium is injected through the annular gap and O_2_ is injected within the high-voltage electrode. In the jet mode, helium is injected within the high-voltage electrode and ambient air is left to fill the annular gap freely. In the *γ* mode, pulse modulation was used to maintain gas temperature near room temperature.

Conditions	Discharge Mode	Applied Power	Treatment Time	Helium Flowrate	O_2_ Flowrate
**Ω**	Ω mode	10 W	4 min	4300 mL min^−1^	0 mL min^−1^
**Ω + O_2_**	Ω mode	10 W	4 min	4300 mL min^−1^	2 mL min^−1^
***γ***	*γ* mode	35 W (100 Hz@20%)	2 min	4300 mL min^−1^	0 mL min^−1^
***γ* + O_2_**	*γ* mode	35 W (100 Hz@20%)	2 min	4300 mL min^−1^	2 mL min^−1^
**Jet**	Jet mode	35 W	10 to 120 s	600 mL min^−1^	0 mL min^−1^

**Figure A1:**
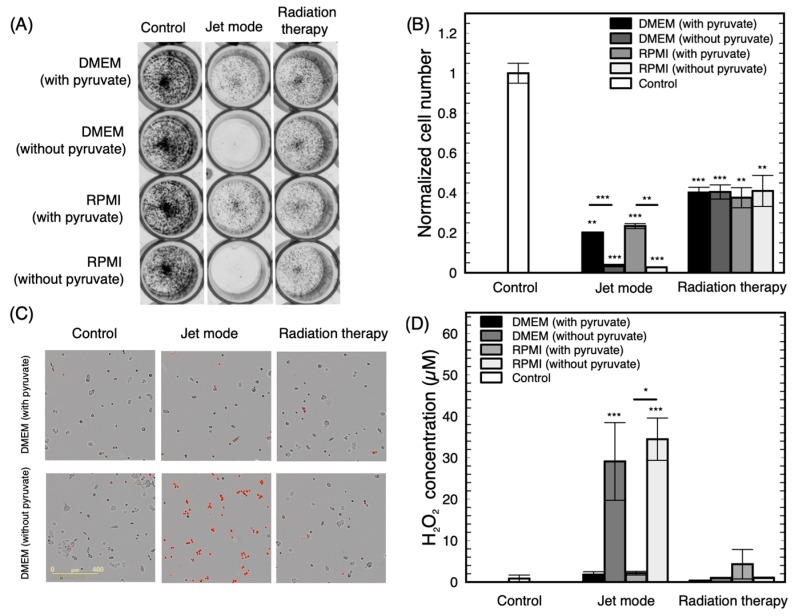
Influence of the culture medium on plasma treatment efficacy for the T47D cell line. (**A**) Representative example of a proliferation assay following plasma treatment (30 s in jet mode) or radiation therapy (4 Gy). (**B**) Quantification of normalized cell numbers from proliferation assays (6 d). (**C**) Fluorescence microscopy after treatments showing dead cells in red (PI). (**D**) H_2_O_2_ concentration measured in the medium after plasma treatment or irradiation. For this figure, n = 3 over independent experiments. * *p* < 0.05, ** *p* < 0.01, *** *p* < 0.001 with respect to the control or other treatment.

## Appendix A

Working with a larger panel of cell lines comes with constraints and one of these is their growth conditions. In this study, according to the supplier recommendations, three different culture media have been used (DMEM, RPMI, and L15). Some cell lines were first treated in their recommended culture medium. However, a substantial difference in sensitivity was found to be associated with the type of medium present during plasma treatment. Figure A1 shows representative results using the T47D cell line to illustrate the differences in plasma sensitivity that were dependent on the medium present during treatment. In proliferation assay, 95% of growth inhibition was measured after a plasma treatment of T47D with RPMI medium, as opposed to 80% when DMEM was used (Figure A1A,B).

Propidium iodide (PI) staining confirmed that rapid cell death was induced when cells were exposed to plasma in RPMI medium (Figure A1C), with almost all cells staining positive for PI. Control or radiation-treated cells were not affected by media composition. Interestingly, when pyruvate, a well-known reactive oxygen species scavenger [63,64], was added to RPMI at the concentration usually found in DMEM, a decrease in plasma cytotoxicity was observed. Pyruvate concentration within the media is a major component affecting the sensitivity of cells to NTP, akin to the suggestion by Babich et al. [65] that pyruvate affects the in vitro cytotoxicity of many other oxidative agents.

High H_2_O_2_ accumulation measured after treatment of pyruvate-free medium could be responsible for this drastic increase of cell death. H_2_O_2_ measurement in media after treatment confirmed this observation. As shown in Figure A1D, the concentration of H_2_O_2_ in the medium after plasma treatment was strongly influenced by the presence of pyruvate. In both RPMI and DMEM, the concentration of H_2_O_2_ dropped from about 40 μM to below the detection level (<1 μM) when pyruvate was present in the medium. From these results, it is clear that the nature of the culture medium present during plasma treatment impacts the sensitivity of cells. Consequently, all cell lines were treated in the same culture medium (DMEM with pyruvate) to avoid this bias. DMEM with pyruvate was chosen to highlight the influence of direct NTP treatment over the capacity of plasma to produce long-term RONS such as H_2_O_2_ [66,67,68,69].

## Appendix B

Using dose response curves of GR values, GR_50_ could be obtained using treatment by the jet mode, RT and olaparib. These values are shown in Table A1. For comparison, both IC_50_ and GR_50_ were calculated for the jet mode. No correlation was observed using Pearson’s correlation test (*p* > 0.5). In addition, no correlation is observed between any of the GR50 and the doubling time using Pearson’s correlation test (*p* > 0.1). Finally, it is also noteworthy that, while the GR50 of RT and jet mode correlates, neither one correlates with the GR50 of olaparib (Pearson’s correlation test with *p* > 0.05), again highlighting the similarity of RT and NTP as physical treatment modalities.

**Table A1:** IC_50_ and GR_50_ obtained by dose response curves for different cell lines. The doubling time of different cell lines is also presented.

Cell Line	IC_50_ of the Jet Mode (s)	GR_50_ of the Jet Mode (s)	GR_50_ of RT (Gy)	GR_50_ of Olaparib (μM)	Doubling Time (d)
MDA-MB-231	1.6	4.9	1.8	9.6	1.5
MDA-MB-468	6.0	10.9	2.3	3.4	1.6
Hs578T	18.4	23.3	4.6	22.3	1.7
HCC1954	6.9	19.2	3.0	18.2	1.7
AU-565	24.1	25.2	3.3	NA	1.8
T47D	9.6	16.0	3.2	17.6	2.1
BT-549	7.8	5.8	2.7	19.2	2.3
MCF-7	15.0	14.1	3.1	4.5	2.3
HCC1569	12.1	13.9	2.1	3.4	2.4
MDA-MB-157	4.6	2.6	1.7	NA	4.1
ZR-75-1	7.9	6.6	0.5	11.4	4.5
MDA-MB-361	31.0	5.1	2.7	5.5	5.4
MDA-MB-175-VII	13.1	4.3	1.0	NA	5.5
HCC1428	48.3	10.2	2.6	2.5	7.7
